# Impact of a Usual Serving Size of Flavanol-Rich Cocoa Powder Ingested with a Diabetic-Suitable Meal on Postprandial Cardiometabolic Parameters in Type 2 Diabetics—A Randomized, Placebo-Controlled, Double-Blind Crossover Study

**DOI:** 10.3390/nu11020417

**Published:** 2019-02-16

**Authors:** Janina Rynarzewski, Lisa Dicks, Benno F. Zimmermann, Birgit Stoffel-Wagner, Norbert Ludwig, Hans-Peter Helfrich, Sabine Ellinger

**Affiliations:** 1 Faculty of Food, Nutrition and Hospitality Sciences, Hochschule Niederrhein, University of Applied Sciences, Rheydter Str. 277, 41065 Mönchengladbach, Germany; ja.rynarzewski@gmail.com (J.R.); Lisa.Dicks@hs-niederrhein.de (L.D.); Norbert.Ludwig@hs-niederrhein.de (N.L.); 2 Department of Nutrition and Food Sciences, University of Bonn, Endenicher Allee 11-13, 53115 Bonn, Germany; benno.zimmermann@uni-bonn.de; 3 Institute of Clinical Chemistry and Clinical Pharmacology, University Hospital Bonn, Sigmund-Freud-Str. 25, 53127 Bonn, Germany; birgit.stoffel-wagner@ukbonn.de; 4 Praxis Anrath, Pastoratstr. 9-11, 47877 Willich, Germany; 5 Institute of Numerical Simulation, University of Bonn, Endenicher Allee 60, 53115 Bonn, Germany; helfrich@uni-bonn.de

**Keywords:** type 2 diabetes, flavanol-rich cocoa, postprandial, meal, glucose metabolism, lipids, blood pressure

## Abstract

Randomized controlled trials indicate that flavanol-rich cocoa intake may improve postprandial glucose and lipid metabolism in patients with type 2 diabetes (T2D), based on studies with meals that impose a strong metabolic load. Hence, the aim of the present study was to investigate whether flavanol-rich cocoa powder ingested as part of a diabetic-suitable meal may beneficially affect glucose, lipid metabolism, and blood pressure (BP) in patients with T2D. Twelve adults with T2D, overweight/obesity, and hypertension ingested capsules with 2.5 g of flavanol-rich cocoa or microcrystalline cellulose with a diabetic-suitable breakfast in a randomized, placebo-controlled, double-blind crossover study. BP was measured and blood samples were taken before, 2 and 4 h after breakfast and capsule intake. Cocoa treatment did not affect glucose, insulin, homeostasis model assessment for insulin resistance (HOMA-IR), triglycerides, total cholesterol, low density lipoprotein-cholesterol, high density lipoprotein-cholesterol, and BP. For glucose, insulin and HOMA-IR, only effects by time were observed after both treatments. Thus, 2.5 g of flavanol-rich cocoa powder ingested as part of a diabetic-suitable meal does not seem to affect postprandial glucose and lipid metabolism and BP in stably-treated diabetics. Nevertheless, future studies with close-meshed investigations are desirable, providing realistic amounts of cocoa together with realistic meals rich in carbohydrates to subjects with T2D or metabolic syndrome, which do not afford pharmacological treatment.

## 1. Introduction

The prevalence of type 2 diabetes (T2D) is increasing globally [1]. In patients with T2D, postprandial hyperglycemia has shown to raise the incidence of cardiovascular disease (CVD) and all-cause mortality [2,3]. Postprandial hyperglycemia is often accompanied by postprandial hypertriglyceridemia, which acts as a further risk factor for CVD. Dietary modifications to lower postprandial glucose and triglyceride values are recommended [4]. Thus, functional food and food ingredients which may improve metabolic and vascular biomarkers could be favorable for patients with T2D [5].

Regular consumption of flavanol-rich cocoa may be beneficial for patients with T2D. Cocoa flavanols have shown to enhance insulin secretion, improve insulin sensitivity in peripheral tissues, lower lipids [6] and increase nitric oxide availability [7]. A decrease in insulin resistance [8], triglycerides [8], low density lipoprotein-cholesterol (LDL-cholesterol) [9], and blood pressure (BP) [8], as well as an increase in insulin sensitivity and high density lipoprotein-cholesterol (HDL-cholesterol) [8] could be observed in meta-analyses of randomized controlled trials (RCTs) after regular cocoa treatment. These effects were even more substantial in morbid subjects than in healthy ones without any comorbidities [8].

In patients with T2D, the postprandial effect of flavanol-rich cocoa on cardiometabolic parameters has only been investigated in a small number of RCTs to date. Most participants received oral hypoglycemic drugs [10,11] as well as lipid- and BP-lowering drugs [11] to ensure stable metabolic and BP control. HDL-cholesterol and endothelial function were improved after 4 h when the fast-food breakfast was ingested together with a flavanol-rich cocoa drink and not with a flavanol-poor placebo drink [11]. Acute hyperglycemia-induced endothelial dysfunction was reduced in individuals with T2D after consumption of flavanol-rich chocolate compared to chocolate low in flavanols [10]. However, a 75-g pure glucose load as provided by Mellor et al. [10] is given as an oral glucose tolerance test and used for diagnostic purposes [12,13]. The fast-food breakfast used by Basu et al. [11] provided 766 kcal, with fat as 59% of total energy. However, meals rich in isolated carbohydrates, saturated fat, and cholesterol and low in dietary fiber are not recommended in T2D [14]. Moreover, 20 g of cocoa powder (about eight tablespoons) for the preparation of a cocoa-rich drink is an unrealistic amount and such a drink is not a typical component of a high-fat-fast-food meal.

Thus, the aim of this study was to investigate whether a usual serving size of flavanol-rich cocoa powder, ingested together with a diabetic-suitable meal, may improve postprandial changes in glucose and lipid metabolism as well as BP.

## 2. Materials and Methods 

### 2.1. Study Design and Intervention

This randomized, placebo-controlled, double-blind crossover study was performed between July and October 2017 in a medical practice (Praxis Anrath, Willich, Germany) according to the Declaration of Helsinki. The study was approved by the ethics committees of the University of Bonn (project identification code: 051/17; date of approval: 22 February 2017) and the Medical Association of North Rhine (project identification code: 2017138; date of approval: 30 May 2017). The trial was registered at German Clinical Trial Register (DRKS-ID: DRKS00012561) on 6 June 2017. Written informed consent was obtained from all participants for inclusion before they participated in the study.

Participants were consecutively recruited and allocated to two different groups by permuted block randomization (block size of four, sequence generated by drawing lots by an uninvolved person). After an overnight fast, both groups ingested five A- and B-capsules, respectively, in different order together with a diabetic-suitable breakfast. Each capsule was filled with 0.5 g ACTICOA™ cocoa powder (Barry Callebaut, Zurich, Switzerland, lot no. 100-F017906-AC-796) or pure microcrystalline cellulose (J. Rettenmaier and Söhne, Rosenberg, Germany) by KP Productions (Koblenz, Germany; certified to ISO 9001/2008, FSSC 22000, and HACCP). Hence, five cocoa-containing capsules provided 2.5 g of cocoa in total, corresponding to one tablespoon and one serving size as recommended by the manufacturer. Both interventions were separated by at least two weeks washout (14–17 days in 75% of all participants; min-max 14–52 days). For both treatments, opaque green capsules of hydroxypropyl methylcellulose were used as they disintegrate and dissolve quickly and completely in the upper gastrointestinal tract [15]. A- and B-capsules were opened after statistical analysis had been finished. Thus, participants as well as researchers were blinded to treatment. The breakfast with A-capsules was ingested at 8.48 ± 0.03 a.m. and the breakfast with B-capsules at 8.47 ± 0.03 a.m. (means ± SEMs), respectively. The breakfast was standardized and consisted of one rye bread roll, 4 g margarine and a homemade avocado spread prepared from 60 g avocado, 1 g lemon juice, and 7 g honey. In case of beneficial effects due to the intake of the cocoa powder, it could be used as valuable ingredient for the avocado spread. The energy and nutrient content of this breakfast is listed in Table 1. 

On both study days, body weight and height, waist and hip circumference as well as body fat mass (FM) were determined in fasting state. Venous blood samples were collected and BP was measured before as well as 2 and 4 h after completing the breakfast and capsules’ intake. All measurements were done by a single trained investigator. The subjects were instructed to maintain their diet on the day before each study day, but to abstain from cocoa products, red wine, green/black tea and fruit/vegetables including juices/nectars. Furthermore, they were advised to take their drugs on both experimental days at the same time as usual to exclude potential confounding effects.

### 2.2. Participants

Twelve patients, aged at least 18 years, suffering from T2D according to the criteria of the World Health Organization (WHO)/International Diabetes Federation (IDF) [16] for at least one year, with dietary or pharmacological treatment, good glucose control (i.e., HbA_1c_ 6.5–7.5%, taking into account individual therapy goals), overweight/obesity (body mass index (BMI) ≥ 25.0 kg/m^2^ [17]) and hypertension (based on the criteria of the European Society of Hypertension/the European Society of Cardiology (ESH/ESC) [18]) were included in the study provided that metabolic control, as ensured by pharmacological treatment, was stable. Insulin therapy, gastrointestinal diseases associated with malabsorption, history of cardiovascular events, pregnancy/lactation, smoking in the last three months, excessive consumption of chocolate (> 100 g/day), red wine (> one glass/day), cocoa drink, green or black tea (> one cup/day), regular use of vitamin preparations or flavanol-rich food supplements (e.g., red wine extract) and drug consumption were exclusion criteria. Eligibility was checked by questionnaire.

### 2.3. Cocoa Powder

ACTICOA™ cocoa powder was used for cocoa treatment, a functional food in which 80% of the natural flavanol content of raw cocoa is preserved due to gentle cocoa processing. According to the product specification, 2.5 g ACTICOA™ cocoa provided 0.6 g protein, 0.4 g fat, and 0.6 g carbohydrates, thus providing 9 kcal (38 kJ) of energy, 52.5 mg theobromine, and 5.0 mg caffeine. The average flavanol content (sum of mono- to decamers) was 8.3% according to the manufacturer (Barry Callebaut). Further details on mono- and oligomeric flavanols were obtained from our own analysis using ultra-high performance liquid chromatography according to the method of Damm et al. [19]. Results on flavanol composition are shown in Table 2. 

### 2.4. Blood Pressure Investigation

BP was measured after a 5 min rest in a seated position in a quiet room as recommended by the ESH/ESC guidelines [18]. For each investigation, three measurements were performed by using a fully automatic BP monitor (OMRON M 500, OMRON Healthcare Europe, Mannheim, Germany) at 1–2 min intervals. The first reading was always discarded as it has been shown to be higher than subsequent readings [20]. A mean value of the second and third reading was calculated and used for statistical analysis.

### 2.5. Blood Sampling

Venous blood was collected into Vacutainer^®^ tubes (Becton Dickinson, Heidelberg, Germany) with sodium fluoride and sodium heparin (for plasma glucose analysis) and in tubes without anticoagulant (for analysis of insulin, triglycerides, total-, LDL- and HDL-cholesterol in serum). All samples were kept in an insulated polystyrene box at room temperature. After collection of the 4-h blood sample, all samples (the fasting one and those obtained 2 and 4 h after the meal) were transported to Niederrhein, University of Applied Sciences, Mönchengladbach, Germany. The whole blood was centrifuged (4 °C, 3000× *g*, 20 min) within 0.5–4.75 h after blood sampling. Glucose, insulin and lipids have shown to remain stable in whole blood up to 24 h at room temperature in Vacutainer^®^ tubes that were also used in our study [21]. Plasma and serum samples were aliquoted and frozen at –80 °C. After completion of the study, they were transported on dry ice to the Institute of Clinical Chemistry and Clinical Pharmacology at the University Hospital Bonn, Germany, for analysis. 

### 2.6. Laboratory Investigations

Glucose, triglycerides, as well as total-, LDL-, and HDL cholesterol were determined by cobas c 701/702 and insulin by cobas e 801 (both Roche/Hitachi, Mannheim, Germany) by means of test kits as described previously [22]. Glucose and insulin were used to calculate homeostasis model assessment for insulin resistance (HOMA-IR) [23]. 

### 2.7. Anthropometric Investigations

Body weight and height were determined on a calibrated digital column scale (seca 910, Hamburg, Germany; accuracy 0.1 kg and 0.1 cm, respectively) to calculate BMI. Waist circumference was measured at the belly button and hip circumference at the maximal circumference (accuracy 0.1 cm) twice with a non-extensible tape. The mean values of both were used to calculate the waist-to-hip ratio (WHR) to determine the body fat distribution according to WHO [24]. FM was determined according to the guidelines of the European Society for Clinical Nutrition and Metabolism (ESPEN) [25]. Resistance and reactance were measured at 800 µA and 50 kHz with the BIA 2000-1 device (Data Input, Pöcking, Germany) as described by Kirch et al. [26]. FM was calculated by using the equation of Kyle et al. [27]. 

### 2.8. Food Intake

Before each study day, food intake, including beverages, was documented in a standardized one-day dietary record. The intake of energy and selected nutrients was determined by Prodi 6.4.0.1 software (Nutri-Science, Freiburg, Germany). 

### 2.9. Statistical Analysis

Statistical analysis was performed by using IBM-SPSS Statistics, version 23 (IBM Crop., Armonk, NY, USA). Metric data were investigated for normal distribution with the Kolmogorov-Smirnov test. Variables without normal distribution were logarithmized. If normal distribution could be assumed, data which were only investigated in fasting state on each study day were compared with each other by using the *t*-test for paired samples. The influence of treatment and time on glucose and lipid metabolism as well as on BP was tested by using repeated-measures ANOVA. In case of significant effects by time, post-hoc tests were carried out. If the variances were homogeneous, Tukey’s test was used. Otherwise, Dunnett’s T3 test was performed. If repeated-measures ANOVA was not applicable due to the lack of normality, changes before and after each treatment were investigated by using Friedman’s test. In case of significant changes, a post-hoc test (two-factorial analysis of variances according to ranks) was done, followed by Wilcoxon signed rank test to compare data of the same points of time between both treatments. *p*-values < 0.05 were considered statistically significant. 

Baseline characteristics of the participants are presented as means ± SDs (metric data) and as frequencies (nominal/ordinal data). Pre- and postintervention data are shown as means ± SEMs and medians (interquartile ranges), respectively. 

## 3. Results

All 12 participants (nine men, three women) completed the study and were included in the statistical analysis. On average, the patients were 68.0 ± 9.0 years old and had been suffering from diabetes for 8.9 ± 5.2 years. Almost two-third of the participants were treated with oral antidiabetic agents, one third with lipid-lowering drugs and all of them with antihypertensiva, mostly providing different active pharmaceutical ingredients (Table 3). Pharmacological treatment did not change during the study. Nutrition status (body weight, BMI, waist circumference, WHR, and FM) was not significantly different between the two study days (Table 4). Furthermore, no significant differences in the intake of energy, nutrients (protein, fat, carbohydrates, dietary fiber, cholesterol, iron, vitamin C) were found on the days before each investigation (Table 4). 

Effects by treatment on glucose metabolism (glucose, insulin, HOMA-IR), lipid metabolism (triglycerides, total cholesterol, LDL-cholesterol, HDL-cholesterol) and on BP could not be observed (Table 5). No interactions between treatment and time occurred. Temporal changes were found for glucose after 2 and 4 h compared to fasting values, irrespective whether the breakfast was ingested together with the capsules providing cocoa (*p* = 0.007) or not (*p =* 0.003). Effects by time could be detected for insulin and HOMA-IR (*p* ≤ 0.001). Insulin and HOMA-IR increased 2 h after both treatments compared to pre-consumption values (insulin: *p =* 0.032 for cocoa treatment; *p =* 0.031 for placebo treatment according to Dunnett’s T3 test; HOMA-IR: *p* < 0.001 for cocoa treatment; *p* = 0.002 for placebo treatment according to Tukey’s test). Afterwards, HOMA-IR decreased significantly (cocoa treatment: *p =* 0.002; placebo treatment: *p* = 0.001; Tukey’s test). Repeated-measures ANOVA revealed effects by time on LDL-cholesterol (*p* = 0.048), LDL/HDL-cholesterol ratio (*p* = 0.004) and on systolic BP (*p* = 0.002), whereas no differences between time points could be detected with Tukey’s test.

Unintended effects by the ingestion of cocoa-containing or cocoa-free capsules together with the meals were not reported.

## 4. Discussion

To the best of our knowledge, this is the first study investigating the postprandial effect of a usual serving-size of a flavanol-rich cocoa powder, provided in addition to a diabetic-suitable meal, on glucose and lipid metabolism and on BP in hypertensive type 2 diabetics with stable metabolism. Contrary to our hypothesis, glucose and lipid metabolism and BP were not influenced by the additional intake of 2.5 g cocoa (Table 5). Only a significant decrease in plasma glucose was observed 4 h vs. 2 h after both treatments. Since the values at 2 h and at 4 h were not significantly different between cocoa and placebo treatment, these changes in plasma glucose might simply reflect the glycemic response induced by the breakfast.

Our results on plasma glucose are similar to those of Mellor et al. [10] and Basu et al. [11]. Both provided a flavanol-rich chocolate 60 min before a glucose challenge (75 g pure glucose) [10] or a flavanol-rich cocoa drink together with a high-fat fast food meal (providing 50 g of carbohydrates, predominantly as starch) [11], and who did not observe any effects either. They also investigated patients who suffered from T2D as well as from overweight or obesity (*n* = 18 [11], *n* = 10 [10]). The patients were similar regarding age and pharmacological treatment (some using oral antidiabetic agents [10,11] as well as lipid- and BP-lowering drugs [11]) to the participants in our study. For cocoa treatment, 13.5 g ACTICOA™ chocolate [10] and a cocoa drink prepared from 20 g cocoa powder [11] were used, respectively. Both studies provided 40 mg epicatechin, which is claimed to be responsible for the vasoprotective effects of cocoa [28], and which in our study was also ingested by 2.5 g cocoa (Table 2). Since the amount of epicatechin in cocoa correlates strongly with the sum of catechin, epicatechin, procyanidins B2, B5, C1, and D (R^2^ = 0.993) [29], the intake of cocoa flavanols in our study and in those of Basu et al. and Mellor et al. were probably comparable. Cocoa flavanols have shown to inhibit digestive enzymes (e.g., α-amylase, α-glucosidase), sodium/glucose cotransporter 1 (SGLT1), dipeptidyl peptidase-IV, and to stimulate incretin secretion (glucagon-like peptide 1, GLP-1; glucose-dependent insulinotropic polypeptide, GIP), which may reduce glycemic response. However, these mechanisms were only investigated in vitro and in animal studies, always using much higher concentrations of flavanols and larger amounts of cocoa than in our study [30].

Regarding insulin and HOMA-IR, we observed only changes by time, but not by treatment. Our result on insulin agrees with the findings of Mellor et al. [10], whereas Basu et al. [11] detected a significant effect of cocoa treatment, leading to higher insulin concentrations and HOMA-IR values at 4 h after cocoa compared to the placebo drink. Cocoa flavanols have been shown to enhance insulin signaling (IRS-1, IRS-2, PI3K-Akt-signaling pathway, AMP kinase) and the translocation of GLUT-4 in vitro and in animals [30]. However, as the dose of epicatechin (40 mg) ingested from cocoa in our study and in those of Basu et al. [11] and Mellor et al. [10] was comparable, ingredients of cocoa other than flavanols may be relevant for changes in insulin secretion and resistance. Brand-Miller et al. [31] observed a higher postprandial insulin secretion in healthy subjects after ingestion of chocolate, cakes, breakfast cereals, ice cream, flavored milk and pudding if these were enriched with cocoa powder. The results of Brand-Miller et al. and Basu et al. may be explained by specific insulinogenic amino acids [11,31]. Their intake might have been much higher in the study of Basu et al. [11] than in our study and in the study of Mellor et al. [10], considering that higher amounts of protein from cocoa (Basu et al.: 2.7 g [11]) were ingested than in our study (0.5 g) (no data available from Mellor et al. [10]). Stearic acid, a fatty acid in cocoa butter, is discussed to stimulate specifically insulin secretion [31]. We provided less cocoa powder (2.5 g) than Basu et al. (20 g) with a lower fat content (16% of dry mass, corresponding to 0.4 g fat compared to Basu et al. [11] (23.5% fat of dry mass, corresponding to 4.7 g; no data on fat content in chocolate available from Mellor et al. [10]). This may further explain the lack of changes in insulin concentration and HOMA-IR by cocoa treatment in our study.

Cocoa treatment affected neither triglycerides nor total, LDL- and HDL-cholesterol in our study. At first glance, this is astonishing as cocoa flavanols may activate catabolic pathways (e.g., β-oxidation) and inhibit anabolic ones (e.g., acetyl CoA carboxylase, HMG-CoA-reductase) in concentrations that can be reached via diet by stimulating 5’-AMP-activated protein kinase [32]. However, the 5’-AMP-activated protein kinase is diminished by insulin through the Akt signaling pathway [33]. Consequently, the insulin response induced by our breakfast might have overwhelmed the effect of cocoa flavanols on 5’-AMP-activated protein kinase. This possibly explains the lack of changes in serum lipids in our study.

Our findings on triglycerides, total and LDL-cholesterol agree with findings of Basu et al. [11], except for HDL-cholesterol which increased after 4 h vs. 1 h if the cocoa drink was ingested with the fast food meal. Since high doses of pure theobromine (850 mg/d) increased HDL-cholesterol in healthy subjects [34], theobromine may be responsible for the increase in HDL-cholesterol, as observed by Basu et al. [11] after cocoa consumption provided 220 mg of theobromine. In our study, the theobromine intake from cocoa (52.5 mg) was possibly too low to induce any beneficial changes in HDL-cholesterol, which may explain the lack of a treatment effect on HDL-cholesterol in our study.

BP and flow-mediated dilation (FMD) could be improved after regular cocoa consumption according to a meta-analysis of RCTs [35]. An increase in FMD was even observed after acute cocoa intake [35]. Hence, a decrease in BP by a cocoa-enriched meal was expected due to an increased arterial elasticity, but was not detectable in our study. However, the increase in FMD after acute cocoa intake was not accompanied by changes in BP in healthy subjects [36] and in subjects with T2D [10,11]. Consequently, FMD seems to be more a sensitive vascular parameter with a stronger response to cocoa intake compared to BP.

Most of our patients were pharmacologically treated for diabetes, lipometabolic disorders, and hypertension (Table 3) as were those of Basu et al. [11] and Mellor et al. [10]. As the cellular and molecular mode of action of cocoa flavanols is partly similar to those of pharmaceuticals, such as metformin and ACE inhibitors (e.g., improving insulin release, secretion and sensitivity in fat, liver tissue, and in muscles [32,37] by increasing the translocation of the GLUT-4 transporter [32], inhibition of the angiotensin converting enzyme [38]), almost no significant effects by cocoa treatment could be achieved. While similar studies on postprandial effects of cocoa with pharmacologically-untreated subjects with T2D would be interesting, these have not become available to date. Gutiérrez-Salmeán et al. have shown that pure (–)-epicatechin (1 mg/kg body weight) ingested within a ready-to-drink oral nutritional supplement rich in carbohydrates (63% of total energy) lowers postprandial glycemic and lipemic response in healthy subjects after 2 and 4 h compared to epicatechin-free treatment, in overweight/obese subjects (*n* = 8) even more so than in normal-weight subjects (*n* = 12) [39]. In another trial, also performed with overweight/obese subjects, cocoa extract (1.4 g, providing 153 mg of epicatechin), in addition to a meal rich in fat and low in carbohydrates (53% and 38% of total energy, respectively) did not change postprandial glucose and lipid metabolism or blood pressure compared to a meal without cocoa extract [40]. In both studies, none of the participants suffered from diabetes and hypertension and did not use glucose, lipid, and BP lowering drugs [39,40]. Thus, in overweight/obese subjects with metabolic disturbances and without pharmacological treatments, postprandial metabolism may be improved by flavanol-rich cocoa as part of a meal rich in low-molecular carbohydrates.

We investigated postprandial changes after 2 and 4 h for the following reasons. First, epicatechin usually achieves maximum concentration in plasma 2 h after cocoa consumption [28]. Second, cardiometabolic effects were observed 2 h [10,11] and 4 h [11] after cocoa ingestion together with a metabolic challenge in type 2 diabetics. Third, beneficial effects in glucose and lipid metabolism were found in obese/overweight subjects 2 and 4 h after a meal if this meal was ingested with pure (–)-epicatechin [39]. However, taking also into account the different plasma kinetics of glucose and insulin in subjects with and without T2D after an oral glucose challenge [41], postprandial changes in glucose metabolism should be investigated in a close-meshed procedure, i.e., every 30–60 min for 2–3 h, to precisely assess the glycemic response in patients with T2D.

The strengths of our RCT include the double-blind, placebo-controlled crossover study design. Moreover, nutritional status and dietary intake were investigated before each treatment. Therefore, confounding effects of lifestyle changes that might have affected our outcome variables are unlikely. The collection of only two blood samples after a meal makes it difficult to assess the postprandial changes in glucose metabolism as precisely as necessary to detect possible differences. This is the major limitation of our study. However, it remains speculative whether this is the reason why we did not detect significant effects by cocoa. As sample size estimation was not possible due to the lack of data, our study might have been underpowered. A larger sample size would allow an adjustment to factors that might be relevant for the response to cocoa. Nevertheless, the results of the present study may be used to calculate the sample size for future studies.

In conclusion, administration of 2.5 g of flavanol-rich cocoa powder together with a diabetic-suitable breakfast does not seem to modulate glucose and lipid metabolism or BP in patients suffering from T2D and hypertension. Nevertheless, future studies are desirable. These should include close-meshed investigations to exactly assess the glycemic and lipemic response in subjects with T2D or metabolic syndrome, but without pharmacological treatment. Moreover, realistic amounts of cocoa should be provided with realistic meals rich in carbohydrates. Additionally, further vascular parameters, such as FMD, should be considered.

## Figures and Tables

**Table 1:** Energy and nutrient content of the breakfast suitable for diabetes.

Ingredients	Energy (kcal)	Protein (g)	Fat (g)	Carbo-Hydrates (g)	Dietary Fiber (g)
Rye bread roll, 60 g ^1^	167	6.3	1.0	31.0	3.4
Margarine, 4 g ^2^	28	0.0	3.0	0.0	–
Avocado, 60 g	83	0.9	8.0	2.0	2.5
Lemon juice, 1 g	0	0.0	0.0	0.0	–
Honey, 7 g ^3^	21	0.0	–	5.0	–
Vanilla flavor, one drop	–	–	–	–	–
∑	299	7.2	12.0	38.0	5.9

Data were calculated by using the nutrition software Prodi (Nutri-Science, Freiburg, Germany). ^1^ Bakery Stinges, Brüggen, Germany; ^2^ Bellasan^®^, Walter Rau Food Factories, Hilter, Germany; ^3^ Goldland^®^, Dr. Krieger’s, Magdeburg, Germany.

**Table 2:** Flavanol composition of the cocoa.

Flavanols	Content per 2.5 g Cocoa Powder
Flavanols, degree of polymerization (per NP-HPLC)	
Monomers (mg)	49.7
Dimers (mg)	13.9
Trimers (mg)	5.5
Tetramers (mg)	4.7
Pentamers (mg)	3.1
Individual flavanols (per RP-HPLC)	
Epicatechin (mg)	40.4
Catechin (mg)	13.6
A-Dimers (mg)	4.3
Procyanidin B2 (mg)	12.3
Procyanidin B5 (mg)	1.3
Procyanidin C1 (mg)	3.1
Trimers ^1^ (mg)	4.1
Trimers ^1^ (mg)	4.4
Procyanidin D (mg)	4.1

^1^ Trimeric procyanidins not further specified. NP-HPLC: normal-phase high-pressure liquid chromatography; RP-HPLC: reversed-phase high-pressure liquid chromatography.

**Table 3:** Demographic and clinical data.

	Participants (*n* = 12)
Demographic data	
Men/women (*n*/*n*)	3/9
Age (years)	68.0 ± 8.7
Diabetes duration (years)	8.9 ± 5.2
Comorbidities *(n)*	
Hypertension	12
Hyperlipidemia	7
Coronary heart disease	3
Vascular diseases	1
Antihyperglycemic drugs *(n)*	7
Glimepiride (sulfonylurea)	4
Gliptine (dipeptidyl peptidase-4 inhibitor)	4
Metformin (biguanide)	7
Repaglinide (meglitinide analogue)	1
Antihypertensive drugs *(n)*	12
Lisinopril (ACE inhibitor)	1
Ramipril (ACE inhibitor)	5
Candesartan (AT1 receptor antagonist)	2
Olmesartan (AT1 receptor antagonist)	2
Valsartan (AT1 receptor antagonist)	1
Amlodipine (calcium channel blocker)	6
Lercanidipine (calcium channel blocker)	1
Bisoprolol (beta-receptor blocker)	3
Carvedilol (beta-receptor blocker)	1
Metoprolol (beta-receptor blocker)	1
Nebivolol (beta-receptor blocker)	2
Hydrochlorothiazide (diuretic)	6
Moxonidine (imidazoline-receptor agonist)	2
Lipid-lowering drugs *(n)*	4
Atorvastatin (statin)	1
Simvastatin (statin)	1
Ezetimibe (NPC1L1 inhibitor)	3

Data are means ± SDs, unless otherwise specified. Results on medication according to individual medication list. The sum of active pharmacological ingredients from antiglycemic, antihypertensive and lipid-lowering drugs exceeded the number of participants as most patients received a combination of different pharmacological ingredients. ACE inhibitor, angiotensin converting enzyme inhibitor; AT1 receptor antagonist, angiotensin II type 1 receptor antagonist; NPC1L1 inhibitor, Niemann-Pick C1-like 1 protein inhibitor.

**Table 4:** Nutrition status in fasting state and nutritional intake on the previous day before each treatment.

	Before Cocoa Treatment (*n* = 12)	Before Placebo Treatment (*n* = 12)	*p*
Nutrition status			
Body weight (kg)	97.4 ± 3.3	98.1 ± 3.0	0.169
BMI (kg/m²)	33.5 ± 0.9	33.7 ± 0.9	0.174
BMI classification ^1^ *(n)*			
Overweight	2	2	–
Obesity	10	10	–
Fat mass (% body weight)	36.3 ± 1.6	36.5 ± 1.6	0.571
Waist circumference (cm)	112.1 ± 2.0	113.0 ± 2.0	0.089
Waist-to-hip ratio	1.0 ± 0.0	1.0 ± 0.0	0.555
Nutritional intake			
Energy (kcal)	1479 ± 149	1675 ± 208	0.354
Protein (g) ^2^	71.8 ± 9.6	80.5 ± 15.2	0.633
Fat (g)	79.5 ± 9.4	81.4 ± 11.7	0.865
Carbohydrates (g)	100.6 ± 11.7	142.7 ± 17.2	0.058
Dietary fiber (g)	10.4 ± 1.4	12.2 ± 1.0	0.365
Cholesterol (mg) ^2^	390.9 ± 87.5	406.2 ± 109.6	0.922
Iron (mg)	7.7 ± 1.0	8.6 ± 1.3	0.582
Vitamin C (mg)	43.4 ± 10.5	44.1 ± 5.1	0.934

*p*-values according to *t*-test for paired samples. ^1^ According to the body mass index classification of the World Health Organization (WHO) (2004). ^2^ Logarithmized values were used for paired *t*-test. Data are means ± SEMs unless indicated otherwise. BMI, body mass index.

**Table 5:** Results on glucose and lipid metabolism and on blood pressure.

	Cocoa Treatment (*n* = 12)			Placebo Treatment (*n* = 12)			Repeated-Measures ANOVA			Friedman Test
	0 h	2 h	4 h	0 h	2 h	4 h	TR	Time	Time x TR	
Glucose metabolism										
Glucose (mmol/L)	7.3 (6.1, 8.3) ^ab^	8.7 (6.9, 9.8) ^a^	6.2 (5.0, 6.6) ^b^	7.5 (6.8, 8.4) ^ab^	9.1 (7.6, 10.6) ^a^	6.5 (5.5, 7.4) ^b^	—	—	—	0.009 ^1^
Insulin (mU/L)	14.2 ± 1.8 ^a^	42.1 ± 7.2 ^b^	22.7 ± 4.3 ^ab^	16.5 ± 1.8 ^a^	40.5 ± 6.1 ^b^	19.8 ± 3.4 ^ab^	0.891	<0.001 ^2^	0.644	—
HOMA-IR (mmol/L) ^3^	4.6 ± 0.6 ^a^	16.7 ± 3.6 ^b^	6.5 ± 1.5 ^a^	5.4 ± 0.6 ^a^	16.8 ± 3.1 ^b^	5.9 ± 1.2 ^a^	0.762	<0.001 ^4^	0.463	—
Lipid status										
Triglycerides (mmol/L)	1.93 ± 0.22	2.03 ± 0.19	1.93 ± 0.19	1.75 ± 0.17	1.96 ± 0.21	2.03 ± 0.23	0.846	0.184	0.280	—
Total cholesterol (mmol/L)	5.01 ± 0.25	5.00 ± 0.25	5.02 ± 0.25	5.05 ± 0.23	4.99 ± 0.25	5.11 ± 0.25	0.904	0.182	0.346	—
LDL-cholesterol (mmol/L)	2.98 ± 0.20 ^a^	2.97 ± 0.20 ^a^	3.01 ± 0.20 ^a^	3.07 ± 0.20 ^a^	3.00 ± 0.20 ^a^	3.08 ± 0.21 ^a^	0.825	0.048 ^4^	0.372	—
HDL-cholesterol (mmol/L)	1.20 ± 0.07	1.19 ± 0.07	1.19 ± 0.07	1.22 ± 0.08	1.18 ± 0.08	1.20 ± 0.08	0.924	0.157	0.473	—
LDL-chol/HDL chol ratio	2.55 ± 0.19 ^a^	2.56 ± 0.19 ^a^	2.59 ± 0.20 ^a^	2.59 ± 0.18 ^a^	2.60 ± 0.18 ^a^	2.64 ± 0.19 ^a^	0.858	0.004 ^4^	0.891	—
Blood pressure										
Systolic (mmHg)	145.2 ± 5.4 ^a^	139.4 ± 5.4 ^a^	138.3 ± 4.7 ^a^	153.0 ± 3.8 ^a^	139.8 ± 4.7 ^a^	139.8 ± 4.3 ^a^	0.591	0.002 ^4^	0.333	—
Diastolic (mmHg)	78.0 ± 2.7	75.2 ± 3.4	77.8 ± 2.8	82.0 ± 2.1	77.9 ± 3.9	78.8 ± 2.5	0.503	0.089	0.600	—

^1^ In case of significant changes, a post-hoc test (two-factorial analysis of variances according to ranks) was done. ^2^ In case of significant effects by time and missing variance homogeneity, Dunnett’s T3 test was performed. ^3^ Logarithmized values were used for repeated-measures ANOVA. ^4^ If effects by time were significant and variance homogeneity was given, Tukey test was performed. Values within the same treatment with different superscript letters differ significantly (*p* ≤ 0.05). Plasma glucose at 2 h and at 4 h was not significantly different between cocoa and placebo treatment (Wilcoxon signed rank test). Data are means ± SEMs and medians (interquartile ranges), respectively. Chol, cholesterol; HDL-cholesterol, high density lipoprotein-cholesterol; HOMA-IR, homeostasis model assessment for insulin resistance; LDL-cholesterol, low density lipoprotein-cholesterol; TR, treatment.

## References

[1] International Diabetes Federation IDF Diabetes Atlas. http://diabetesatlas.org.

[2] Cavalot F., Pagliarino A., Valle M., Di Martino L., Bonomo K., Massucco P., Anfossi G., Trovati M. (2011). Postprandial blood glucose predicts cardiovascular events and all-cause mortality in type 2 diabetes in a 14-year follow-up: Lessons from the San Luigi Gonzaga Diabetes Study. Diabetes Care.

[3] Takao T., Suka M., Yanagisawa H., Iwamoto Y. (2017). Impact of postprandial hyperglycemia at clinic visits on the incidence of cardiovascular events and all-cause mortality in patients with type 2 diabetes. J. Diabetes Investig..

[4] Pappas C., Kandaraki E.A., Tsirona S., Kountouras D., Kassi G., Diamanti-Kandarakis E. (2016). Postprandial dysmetabolism: Too early or too late?. Hormones (Athens).

[5] Alkhatib A., Tsang C., Tiss A., Bahorun T., Arefanian H., Barake R., Khadir A., Tuomilehto J. (2017). Functional foods and lifestyle approaches for diabetes prevention and management. Nutrients.

[6] Ramos S., Martín M.A., Goya L. (2017). Effects of cocoa antioxidants in type 2 diabetes mellitus. Antioxidants (Basel).

[7] Ludovici V., Barthelmes J., Nagele M.P., Enseleit F., Ferri C., Flammer A.J., Ruschitzka F., Sudano I. (2017). Cocoa, blood pressure, and vascular function. Front. Nutr..

[8] Lin X., Zhang I., Li A., Manson J.E., Sesso H.D., Wang L., Liu S. (2016). Cocoa flavanol intake and biomarkers for cardiometabolic health: A systematic review and meta-analysis of randomized controlled trials. J. Nutr..

[9] Tokede O.A., Gaziano J.M., Djousse L. (2011). Effects of cocoa products/dark chocolate on serum lipids: A meta-analysis. Eur. J. Clin. Nutr..

[10] Mellor D.D., Madden L.A., Smith K.A., Kilpatrick E.S., Atkin S.L. (2013). High-polyphenol chocolate reduces endothelial dysfunction and oxidative stress during acute transient hyperglycaemia in type 2 diabetes: A pilot randomized controlled trial. Diabet. Med..

[11] Basu A., Betts N.M., Leyva M.J., Fu D., Aston C.E., Lyons T.J. (2015). Acute cocoa supplementation increases postprandial HDL cholesterol and insulin in obese adults with type 2 diabetes after consumption of a high-fat breakfast. J. Nutr..

[12] American Diabetes Association (2019). 2. Classification and diagnosis of diabetes: Standards of medical care in diabetes-2019. Diabetes Care.

[13] Kerner W., Bruckel J., German Diabetes Association (2014). Definition, classification and diagnosis of diabetes mellitus. Exp. Clin. Endocrinol. Diabetes.

[14] Evert A.B., Boucher J.L. (2014). New diabetes nutrition therapy recommendations: What you need to know. Diabetes Spectr..

[15] Al-Tabakha M.M. (2010). HPMC capsules: Current status and future prospects. J. Pharm. Pharm. Sci..

[16] World Health Organization (WHO), International Diabetes Federation (IDF) (2006). Definition and Diagnosis of Diabetes Mellitus and Intermediate Hyperglycaemia—Report of a WHO/IDF Consultation.

[17] World Health Organization (2000). Obesity: Preventing and Managing the Global Epidemic—Report of a WHO Consultation (WHO Technical Report Series 894).

[18] Mancia G., Fagard R., Narkiewicz K., Redon J., Zanchetti A., Bohm M., Christiaens T., Cifkova R., De Backer G., Dominiczak A. (2013). 2013 ESH/ESC guidelines for the management of arterial hypertension: The Task Force for the Management of Arterial Hypertension of the European Society of Hypertension (ESH) and of the European Society of Cardiology (ESC). Eur. Heart J..

[19] Damm I., Enger E., Chrubasik-Hausmann S., Schieber A., Zimmermann B.F. (2016). Fast and comprehensive analysis of secondary metabolites in cocoa products using ultra high-performance liquid chromatography directly after pressurized liquid extraction. J. Sep. Sci..

[20] Pickering T.G., Hall J.E., Appel L.J., Falkner B.E., Graves J.W., Hill M.N., Jones D.H., Kurtz T., Sheps S.G., Roccella E.J. (2005). Recommendations for blood pressure measurement in humans: An AHA scientific statement from the Council on High Blood Pressure Research Professional and Public Education Subcommittee. J. Clin. Hypertens. (Greenwich).

[21] Oddoze C., Lombard E., Portugal H. (2012). Stability study of 81 analytes in human whole blood, in serum and in plasma. Clin. Biochem..

[22] Dicks L., Kirch N., Gronwald D., Wernken K., Zimmermann B.F., Helfrich H.P., Ellinger S. (2018). Regular intake of a usual serving size of flavanol-rich cocoa powder does not affect cardiometabolic parameters in stably treated patients with type 2 diabetes and hypertension—A double-blinded, randomized, placebo-controlled trial. Nutrients.

[23] Matthews D.R., Hosker J.P., Rudenski A.S., Naylor B.A., Treacher D.F., Turner R.C. (1985). Homeostasis model assessment: Insulin resistance and beta-cell function from fasting plasma glucose and insulin concentrations in man. Diabetologia.

[24] World Health Organization (2011). Waist Circumference and Waist-Hip Ratio: Report of a WHO Expert Consultation.

[25] Kyle U.G., Bosaeus I., De Lorenzo A.D., Deurenberg P., Elia M., Manuel Gomez J., Lilienthal Heitmann B., Kent-Smith L., Melchior J.C., Pirlich M. (2004). Bioelectrical impedance analysis-part II: Utilization in clinical practice. Clin. Nutr..

[26] Kirch N., Berk L., Liegl Y., Adelsbach M., Zimmermann B.F., Stehle P., Stoffel-Wagner B., Ludwig N., Schieber A., Helfrich H.P. (2018). A nutritive dose of pure (−)-epicatechin does not beneficially affect increased cardiometabolic risk factors in overweight-to-obese adults—A randomized, placebo-controlled, double-blind crossover study. Am. J. Clin. Nutr..

[27] Kyle U.G., Genton L., Karsegard L., Slosman D.O., Pichard C. (2001). Single prediction equation for bioelectrical impedance analysis in adults aged 20–94 years. Nutrition.

[28] Kirch N., Ellinger S. (2014). Cocoa flavanols and cardioprotective effects. What flavanols may contribute to vascular health?. Ernahrungs Umschau.

[29] Cooper K.A., Campos-Gimenez E., Jimenez Alvarez D., Nagy K., Donovan J.L., Williamson G. (2007). Rapid reversed phase ultra-performance liquid chromatography analysis of the major cocoa polyphenols and inter-relationships of their concentrations in chocolate. J. Agric. Food Chem..

[30] Strat K.M., Rowley T.J.T., Smithson A.T., Tessem J.S., Hulver M.W., Liu D., Davy B.M., Davy K.P., Neilson A.P. (2016). Mechanisms by which cocoa flavanols improve metabolic syndrome and related disorders. J. Nutr. Biochem..

[31] Brand-Miller J., Holt S.H., de Jong V., Petocz P. (2003). Cocoa powder increases postprandial insulinemia in lean young adults. J. Nutr..

[32] Martin M.A., Goya L., Ramos S. (2016). Antidiabetic actions of cocoa flavanols. Mol. Nutr. Food Res..

[33] Valentine R.J., Coughlan K.A., Ruderman N.B., Saha A.K. (2014). Insulin inhibits AMPK activity and phosphorylates AMPK Ser(4)(8)(5)/(4)(9)(1) through Akt in hepatocytes, myotubes and incubated rat skeletal muscle. Arch. Biochem. Biophys..

[34] Neufingerl N., Zebregs Y.E., Schuring E.A., Trautwein E.A. (2013). Effect of cocoa and theobromine consumption on serum HDL-cholesterol concentrations: A randomized controlled trial. Am. J. Clin. Nutr..

[35] Hooper L., Kay C., Abdelhamid A., Kroon P.A., Cohn J.S., Rimm E.B., Cassidy A. (2012). Effects of chocolate, cocoa, and flavan-3-ols on cardiovascular health: A systematic review and meta-analysis of randomized trials. Am. J. Clin. Nutr..

[36] Westphal S., Luley C. (2011). Flavanol-rich cocoa ameliorates lipemia-induced endothelial dysfunction. Heart Vessels.

[37] Cordero-Herrera I., Martín M.A., Bravo L., Goya L., Ramos S. (2013). Cocoa flavonoids improve insulin signalling and modulate glucose production via AKT and AMPK in HepG2 cells. Mol. Nutr. Food Res..

[38] Bernstein K.E. (2006). Views of the renin-angiotensin system: Brilling, mimsy, and slithy tove. Hypertension.

[39] Gutiérrez-Salmeán G., Ortiz-Vilchis P., Vacaseydel C.M., Rubio-Gayosso I., Meaney E., Villarreal F., Ramírez-Sánchez I., Ceballos G. (2014). Acute effects of an oral supplement of (−)-epicatechin on postprandial fat and carbohydrate metabolism in normal and overweight subjects. Food Funct..

[40] Ibero-Baraibar I., Suárez M., Arola-Arnal A., Zulet M.A., Martinez J.A. (2016). Cocoa extract intake for 4 weeks reduces postprandial systolic blood pressure response of obese subjects, even after following an energy-restricted diet. Food Nutr. Res..

[41] Kramer C.K., Vuksan V., Choi H., Zinman B., Retnakaran R. (2014). Emerging parameters of the insulin and glucose response on the oral glucose tolerance test: Reproducibility and implications for glucose homeostasis in individuals with and without diabetes. Diabetes Res. Clin. Pract..

